# Epidemiologic Analysis of Efforts to Achieve and Sustain Malaria Elimination along the China–Myanmar Border

**DOI:** 10.3201/eid2711.204428

**Published:** 2021-11

**Authors:** Fang Huang, Li Zhang, Hong Tu, Yan-Wen Cui, Shui-Sen Zhou, Zhi-Gui Xia, Hong-Ning Zhou

**Affiliations:** National Institute of Parasitic Diseases, Chinese Center for Disease Control and Prevention (Chinese Center for Tropical Diseases Research), Shanghai, China (F. Huang, L. Zhang, H. Tu, Y.-W. Cui, S.-S. Zhou, Z.-G. Xia);; NHC Key Laboratory of Parasite and Vector Biology, Shanghai (F. Huang, L. Zhang, H. Tu, Y.-W. Cui, S.-S. Zhou, Z.-G. Xia);; World Health Organization Collaborating Centre for Tropical Diseases, Shanghai (F. Huang, L. Zhang, H. Tu, Y.-W. Cui, S.-S. Zhou, Z.-G. Xia);; National Center for International Research on Tropical Diseases, Shanghai, China (F. Huang, L. Zhang, H. Tu, Y.-W. Cui, S.-S. Zhou, Z.-G. Xia);; Yunnan Institute of Parasitic Diseases, Puer, China (H.-N. Zhou)

**Keywords:** malaria, vector-borne infections, China, Myanmar

## Abstract

Malaria cases have dramatically declined in China along the Myanmar border, attributed mainly to adoption of the 1-3-7 surveillance and response approach. No indigenous cases have been reported in China since 2017. Counties in the middle and southern part of the border area have a higher risk for malaria importation and reestablishment after elimination.

In 2010, China issued the National Malaria Elimination Action Plan (2010–2020), with the goal of eliminating malaria nationwide by 2020 (*1*). Malaria cases in China subsequently decreased dramatically, and no indigenous cases have been reported since 2017 (*2*). In 2020, on the basis of successful subnational verification, China submitted an official request to the World Health Organization for certification of national malaria elimination, which requires a country to provide evidence that local malaria transmission has been fully interrupted, that zero indigenous human malaria cases have been reported for >3 consecutive years, and that an adequate program for preventing reestablishment of indigenous transmission is fully functional throughout the country (*3*). However, the China–Myanmar border of Yunnan Province has attracted considerable attention because of a substantial risk of reintroduction of malaria from bordering areas of Myanmar (*4*). This border region is extremely remote, has high rates of poverty, and is inhabited by local minority nationalities (*5*,*6*). A total of 18 counties in Yunnan Province share the 1,997-km border with the townships of Myanmar in which malaria is endemic; the border provides no natural barriers and poses a high risk for malaria reintroduction into China.

## The Study

We extracted data on reported malaria cases and foci during 2013–2019 from the web-based China Information System for Disease Control and Prevention and comprised data from passive case detection, reactive case detection among foci residents and case co-travelers, and proactive case detection among at-risk populations. Indigenous cases were defined as cases in patients who contracted malaria by bites from *Anopheles* mosquitoes within China without direct link to transmission from an imported case, whereas imported cases were defined as cases in patients who had exposure history in any malaria-endemic areas abroad before the onset of illness (*7*,*8*). *Plasmodium* spp. were determined by microscopy or rapid diagnosis test and PCR (*8*). This study was an epidemiologic analysis of malaria along the China–Myanmar border to identify the risk for malaria reestablishment in the stage after elimination.

During 2013–2019, a total of 2,222 malaria cases were reported from the 18 border counties; 1 death occurred. Total cases fell from 465 in 2013 to 148 in 2019; indigenous cases dropped to zero by 2017, and the number of imported cases also declined (Table 1). This decrease was mainly attributed to the extensive adoption of the 1-3-7 approach to surveillance and response: case reporting within 1 day, investigation within 3 days, and focus investigation and response within 7 days. Case-patients ranged in age from 19 to 59 years, and men and outdoor workers were at considerably higher risk of contracting malaria (p<0.0001) (Table 1). 

**Table 1:** Demographic characteristics of reported malaria cases in the 18 counties in China along the border with Myanmar, 2013–2019*

Characteristics	No. cases by year								No. cases by type		Total cases	p value†
2013	2014	2015	2016	2017	2018	2019	Imported	Indigenous
Total cases	465	392	478	312	263	164	148		2,130	92		
Sex												
M	392	349	404	231	179	124	109		1,726	62	1,788	0.0012
F	73	43	74	81	84	40	39		404	30	434	
Age group, y												
<5	3	9	9	9	10	5	1		41	5	46	
5–18	42	20	36	35	37	19	7		176	20	196	<0.0001
19–59	415	357	423	253	202	127	132		1,850	59	1,909	
>60	5	6	10	15	14	13	8		63	8	71	
Occupation‡												
Outdoor worker	371	323	323	235	160	95	85		1,536	56	1,592	
Indoor worker	37	28	81	10	13	15	21		204	1	205	<0.0001
Unclear	50	35	51	56	84	53	42		348	23	371	
Missing	7	6	23	11	6	1	0		42	12	54	
*Plasmodium* spp.												
* P. vivax*	379	330	428	291	253	151	146		1,900	78	1,978	
* P. falciparum*	80	60	48	21	10	9	2		217	13	230	
* P. malariae*	0	2	1	0	0	1	0		4	0	4	0.4653
* P. falciparum + P. vivax*	6	0	1	0	0	0	0		6	1	7	
* P. falciparum + P. ovale*	0	0	0	0	0	3	0		3	0	3	
Destination of oversea travel and species												
Myanmar											2,056	
* P. falciparum*	71	55	46	16	9	6	1		204	NA		
* P. vivax*	321	294	399	283	246	150	146		1,839	NA		
Other species	5	2	2	0	0	4	0		13	NA		
Other countries											74	
* P. falciparum*	2	0	1	5	1	3	1		13	NA		
* P. vivax*	19	8	19	7	7	1	0		61	NA		

*NA, not available. †The number of cases over years were compared by using a χ^2^ or Fisher exact test according to sample size (>5 or <5) by SAS software. ‡Outdoor workers are persons whose activity is mostly conducted outside, including architectural engineers, construction workers, farmers, fishermen, overseas migrant workers (expatriate Chinese nationals), open mine workers, sailors or truck drivers, field engineers, herdsmen, military or soldiers, etc. Indoor workers include businessmen, caterers, interpreters, medical staff, office workers, teachers, actors, flight attendants, babysitters, middlemen, cooks, diplomats, financial staff, journalists, underground mine workers, prisoners (although not a worker per se, prisoners were officially classified as an indoor worker since their time is spent indoors), researchers, waiters, etc. Unclear indicates those for whom risk exposure cannot clearly be estimated, including children, retirees, self-employed persons, students, unemployed persons, athletes, tourists, etc. Missing data were not included in the statistical analysis.

**Figure 1:**
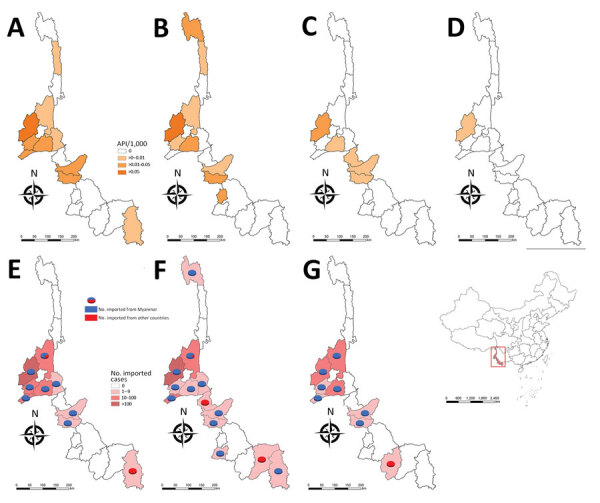
API per 1,000 persons and numbers of imported cases by year in the 18 China counties along the border with Myanmar, 2013–2019. A) 2013 API, B) 2014 API, C) 2015 API, D) 2016 API; E) 2017 imported cases, F) 2018 imported cases, G) 2019 imported cases. Inset map shows location of China–Myanmar border area (rectangle). API, annual parasite index.

In 2013, malaria cases reported from the 18 border counties accounted for 80.6% of total cases in Yunnan Province; 89.9% (418/465) were imported cases. Indigenous cases (10.1%, 47/465) were distributed in 10 border counties (Figure 1). Yingjiang County reported 38.3% (18/47) of total indigenous cases, along with the highest annual parasite index of 0.058. Five counties displayed an annual parasite index range of 0.01–0.05 (Figure 1). The number of counties reporting indigenous cases decreased from 10 in 2013 to 1 in 2016 (Figure 1). The last indigenous *P. falciparum* case in China was in Cangyuan County in 2015 and the last indigenous *P. vivax* case in Yingjiang County in 2016. Most imported malaria cases were distributed in the middle part of the borderline, especially Yingjiang and Tengchong Counties (Figure 1); 96.5% (2,056/2,130) of total imported cases in the 18 border counties were from Myanmar (Table 1). During 2017–2019, a total of 97.7% (562/575) of imported cases and 98.5% (542/550) of *P. vivax* cases were from Myanmar (Table 1). The counties bordering areas of Myanmar, where rates of malaria were highest, displayed higher numbers of imported cases (*9*).

**Figure 2:**
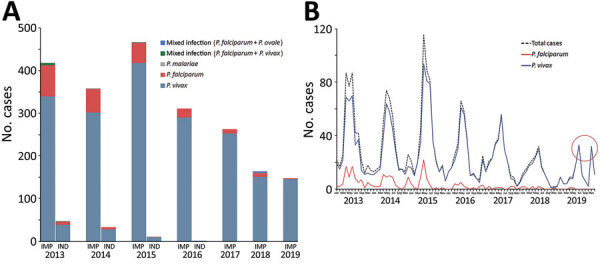
Malaria cases in the 18 counties in China along the border with Myanmar, 2013–2019. A) Proportions of *Plasmodium* species cases. B) Monthly reported malaria cases. Red circle highlights the double peaks identified in July and November 2019. IMP, imported; IND, indigenous.

*P. vivax* was the predominant species. *P. vivax* cases accounted for 95.7% of total reported cases during 2017–2019, whereas the proportion of *P. falciparum* declined to 1.4% (Figure 2, panel A). Four cases of *P. malariae* and 10 cases of mixed infections were reported; no *P. ovale* cases were reported. A total of 43 relapsing cases (*P. vivax* cases that recurred 1 month later with neither evidence of an epidemiologic link to additional cases nor as a result of incomplete clearance of original asexual parasites) were reported during 2013–2019, which indicates the need for adherence to the full primaquine regimen and possible resistance to the drug for eliminating the hypnozoites. 

The malaria transmission peak was from April to August; another slight peak occurred from December to the following January (Figure 2, panel B). This transmission coincided with the local natural environment and was strongly correlated with the abundance of *Anopheles* spp. mosquitoes. Of note, double peaks were identified in July and November 2019. The first peak was postponed, whereas the second peak shifted 1 month earlier. This change was primarily caused by migration in the local population. The temporal distribution pattern of *P. vivax* and *P. falciparum* was different (Figure 2, panel B) because *P. falciparum* cases were few and most were imported, mainly because of population movement and migration.

The median interval between onset of illness and diagnosis of malaria varied widely (range 2–10 days; Table 2), because the capability for diagnosis in some healthcare facilities was relatively low; training is needed to strengthen case detection and diagnosis capabilities. In addition, this range reflects the knowledge, attitudes, and practice gaps regarding malaria treatment-seeking of residents. The median interval between diagnosis and treatment was 0 days, except for in Ximeng County, which had a median interval of 0.5 days (Table 2). These rates indicate the capacity of hospital response was strong. The 1-3-7 approach was adopted nationally in China in early 2012 (*10*). During 2013–2019, all malaria cases were reported within 1 day, 95.6% of cases were investigated within 3 days, and in 8 of 18 counties 100% of cases were investigated within 3 days in all years studied. Longchuan, Gengma, and Yingjiang Centers for Disease Control and Prevention took >3 days to complete investigation of cases from remote areas. Malaria focus in China is defined as the circumscribed village or community with a reported case (*11*). During 2013–2019, a total of 97.9% (381/389) of foci were investigated and responded to within 7 days in 10 counties (Table 2). Depending on the nature of the focus and its state of transmission, the corresponding response actions were selected; these actions consisted of indoor residual spraying, reactive case detection, case treatment, and health education (*7*). No secondary cases have been reported because of the prompt and targeted interventions in all the foci.

**Table 2:** Characteristics of implementation of the 1-3-7 approach to malaria surveillance and response in 18 counties in China along the border with Myanmar, 2013–2019*

County	No. reported cases	Days from illness onset to diagnosis, median (IQR)	Days from diagnosis to treatment, median (IQR)	Case reported within 1 d, %	Case investigated within 3 d, %	Foci response within 7 d, %
Zhenkang	6	4.5 (4–7.3)	0.0 (0–0.75)	100.0	100.0	–
Menghai	3	10.0 (6–12)	0.0 (0–0.5)	100.0	100.0	–
Lancang	9	7.0 (3–11)	0.0 (0)	100.0	100.0	–
Jinghong	14	5.0 (3–6)	0.0 (0)	100.0	92.9	–
Gengma	29	3.0 (1–6)	0.0 (0–1)	100.0	82.8	100.0 (4/4)
Mengla	30	2.0 (1–5)	0.0 (0–1)	100.0	93.3	100.0 (1/1)
Menglian	20	3.5 (0–7.5)	0.0 (0–2.25)	100.0	100.0	100.0 (1/1)
Lushui	18	6.5 (3–10)	0.0 (0)	100.0	100.0	–
Ximeng	4	4.5 (3.8–6)	0.5 (0–1)	100.0	100.0	–
Fugong	11	4.0 (3–6.5)	0.0 (0)	100.0	100.0	–
Cangyuan	35	4.0 (2–7.5)	0.0 (0)	100.0	97.1	–
Longchuan	69	2.0 (1–4)	0.0 (0–1)	100.0	82.6	100.0 (20/20)
Longling	75	3.0 (2–5)	0.0 (0)	100.0	98.7	85.7 (6/7)
Gongshan	5	4.0 (1–5)	0.0 (0–5)	100.0	100.0	100.0 (1/1)
Mangshi	112	3.0 (1–4)	0.0 (0–2)	100.0	92.0	96.2 (25/26)
Tengchong	525	2.0 (1–4)	0.0 (0–2)	100.0	95.6	98.0 (48/49)
Yingjiang	895	2.0 (1–4)	0.0 (0)	100.0	88.4	98.5 (256/261)
Ruili	362	2.5 (1–5)	0.0 (0)	100.0	97.2	100.0 (19/19)

*IQR, interquartile range, –, no focus reported.

## Conclusions

China has set a goal to eliminate malaria by 2020, and Myanmar has set a goal to eliminate malaria by 2030 (*1*,*12*). This study demonstrated that local malaria transmission has been interrupted in Yunnan Province at the China–Myanmar border, although the risk for malaria reintroduction still exists. The complex geographic conditions and large migrant population along the border, in addition to reservoirs of symptomatic and asymptomatic infection in neighboring Myanmar (*13*), are obstacles to consolidating achievements in malaria elimination (*5*,*14*). Another noteworthy factor is the coronavirus disease pandemic. Maintaining full engagement with malaria control is challenging given the simultaneous demands of the pandemic (*15*).

In summary, malaria elimination has been achieved in the counties in China along the border with Myanmar. However, continued strong surveillance, multisectoral collaboration, and cross-border cooperation are of high priority to reduce the risk for malaria reintroduction and sustain its elimination.
